# Thermodynamic Basis of Molecular Diffusion through Cyanobacterial Septal Junctions

**DOI:** 10.1128/mBio.00529-17

**Published:** 2017-05-30

**Authors:** Jonghoon Kang, Courtney N. Burten, Gihun Hong

**Affiliations:** Department of Biology, Valdosta State University, Valdosta, Georgia, USA; Johns Hopkins Bloomberg School of Public Health

**Keywords:** diffusion, kinetics, membrane transport, thermodynamics

## LETTER

A recent paper published by Nieves-Morión and colleagues proposes that the intercellular transfer of small molecules in heterocyst-forming cyanobacteria (calcein, 5-carboxyfluorescein [5-CF], and esculin) is mediated by simple diffusion based on the *Q*_10_ values of the kinetics (1). In this letter, we show our analysis of their results to provide a thermodynamic basis of the kinetics.

**FIG 1:**
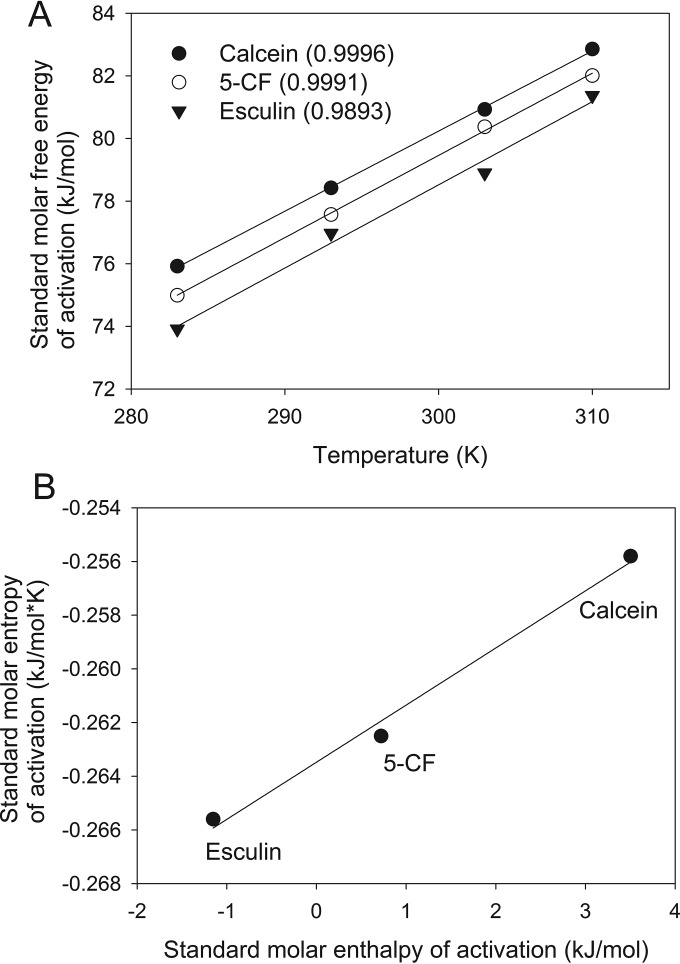
(A) Temperature-dependent standard molar free energy of activation for the intercellular transfer of calcein, 5-carboxyfluorescein (5-CF), and esculin. *R*^2^ of the fitting is shown in the figure (three values in parentheses). (B) Relationship between enthalpy and entropy of activation (*R*^2^ = 0.9907). SigmaPlot (version 11; Systat Software, Inc., San Jose, CA) was used for graph preparation and statistical analysis.

We obtained rate constant values of the intercellular transfer for those molecules from Fig. 1 of the original paper (1) using the ImageJ program (https://imagej.nih.gov/ij/). We calculated molar free energy of activation (Δ*G*_*a*_°) for each molecule at each temperature (*T*) using the Eyring equation (equation 1) from the corresponding rate constant (*k*):

(1) $$\Delta G_{a}{}^{\circ}=-RT\ln\frac{kh}{k_{B}T}$$

where *R*, *h*, and *k*_*B*_ are the ideal gas constant (8.3145 J/K mol), Planck’s constant (6.6261 × 10^−34^ J s), and the Boltzmann constant (1.3807 × 10^−23^ J/K), respectively (2). Δ*G*_*a*_° for each molecule shows a strong linear relationship with temperature (Fig. 1A). From this relationship, we obtained molar enthalpy and entropy of activation (Δ*H*_*a*_° and Δ*S*_*a*_°) for the process using the following equation:

(2) $$\Delta G_{a}{}^{\circ}=\Delta H_{a}{}^{\circ}-T\Delta S_{a}{}^{\circ}$$

Figure 1B shows there is a correlation between Δ*H*_*a*_° and Δ*S*_*a*_° for these three molecules. This correlation is called enthalpy-entropy compensation, suggesting that a common mechanism is responsible for the intercellular transfer of these three molecules (3). Furthermore, Δ*S*_*a*_° was found to be the major component in Δ*G*_*a*_° for all three molecules, as it explains at least 95% of the value of Δ*G*_*a*_° at 37°C. This indicates that the intercellular transfer process is highly unfavorable in terms of entropy. This feature correlates with the suggestion made by the original paper in that the simple diffusion of molecules through a restricted space reduces the entropy of the molecules because entropy is proportional to the volume available (2).

Membrane transport kinetics is typically analyzed in terms of activation energy (*E*_*a*_). Here we obtained the *E*_*a*_ values for all three molecules from Δ*H*_*a*_° using the following equation:

(3) $$E_{a}=\Delta H_{a}{}^{\circ}+RT$$

The *E*_*a*_ values at 37°C are 6.1, 3.3, and 1.4 kJ/mol for calcein, 5-CF, and esculin, respectively. These values are much smaller than that of typical facilitated diffusion. For example, facilitated diffusion for glucose uptake has an *E*_*a*_ of 64 kJ/mol (4). The values of *E*_*a*_ are comparable to the hydrogen bonding energy (4 to 40 kJ/mol) (2), suggesting that dissociation of water molecules from the molecules is involved in the diffusion. Our thermodynamic analysis confirms that the intercellular transfer of those molecules is mediated by simple diffusion.

After data analyses were completed, it was found that there was a relationship between both molecular weight and charge of a molecule with the molecule’s entropy and enthalpy values (Table 1). We were unable to conclude which of the structural parameters is responsible for the correlation of the thermodynamic parameters because of the inherent correlation between those two structural parameters themselves. Calcein is a relatively large molecule possessing a charge of −5, while esculin is a small molecule with no charge and 5-CF is of moderate size and possess a charge of −1. Therefore, we suggest a future experiment in which intercellular transfer is measured with small molecules possessing a high negative charge and/or large molecules with a low negative charge to identify which factor is responsible for the correlation to the thermodynamic parameters. This will greatly enhance understanding of the process.

**TABLE 1:** Pearson correlation coefficient values between thermodynamic and structural parameters

Parameter	Pearson correlation coefficient value between parameter:			
	Δ*H*_*a*_°	Δ*S*_*a*_°	Mol wt	Negative charge
Δ*H*_*a*_°	1	0.995	0.957	0.976
Δ*S*_*a*_°		1	0.981	0.992
Mol wt			1	0.997
Negative charge				1
